# Efficient Epstein-Barr Virus Progeny Production Mediated by Cancer-Derived LMP1 and Virally-Encoded microRNAs

**DOI:** 10.3390/microorganisms7050119

**Published:** 2019-04-30

**Authors:** Misako Yajima, Mamiko Miyata, Kazufumi Ikuta, Yasuhisa Hasegawa, Chitose Oneyama, Teru Kanda

**Affiliations:** 1 Division of Microbiology, Faculty of Medicine, Tohoku Medical and Pharmaceutical University, Sendai 983-8536, Japan; yajima@tohoku-mpu.ac.jp (M.Y.); ikutak@tohoku-mpu.ac.jp (K.I.); 2 Division of Cancer Cell Regulation, Aichi Cancer Center Research Institute, Nagoya 464-8681, Japan; mmiyata@aichi-cc.jp (M.M.); coneyama@aichi-cc.jp (C.O.); 3 Department of Head and Neck Surgery, Aichi Cancer Center Hospital, Nagoya 464-8681, Japan; hasegawa@murakami.asahi-u.ac.jp; 4 Department of Head and Neck Surgery, Asahi University Hospital, Gifu 500-8523, Japan

**Keywords:** Epstein-Barr virus, Nasopharyngeal carcinoma, LMP1, BART, microRNA

## Abstract

Epstein-Barr virus (EBV) genomes, particularly their latent genes, are heterogeneous among strains. The heterogeneity of EBV-encoded latent membrane protein 1 (LMP1) raises the question of whether there are functional differences between LMP1 expressed by cancer-associated EBV and that by non-cancerous strains. Here, we used bacterial artificial chromosome (BAC)-cloned EBV genomes retaining all virally encoded microRNA (miRNA) genes to investigate the functions of cancer-derived LMP1 in the context of the EBV genome. HEK293 cells were stably transfected with EBV-BAC clone DNAs encoding either nasopharyngeal carcinoma (NPC)-derived CAO-LMP1 (LMP1_CAO_) or LMP1 from a prototype B95-8 strain of EBV (LMP1_B95-8_). When an EBV-BAC clone DNA encoding LMP1_CAO_ was stably transfected into HEK293 cells, it generated many more stable transformants than the control clone encoding LMP1_B95-8_. Furthermore, stably transfected HEK293 cells exhibited highly efficient production of progeny virus. Importantly, deletion of the clustered viral miRNA genes compromised the ability to produce progeny viruses. These results indicate that cancer-derived LMP1 and viral miRNAs together are necessary for efficient production of progeny virus, and that the resulting increase in efficiency contributes to EBV-mediated epithelial carcinogenesis.

## 1. Introduction

Epstein-Barr virus (EBV) is an oncogenic herpesvirus that causes various lymphoproliferative diseases, including B cell lymphomas and T/NK lymphomas [1]. EBV also plays a role in the development of epithelial cancers, such as nasopharyngeal carcinoma (NPC) and gastric cancer [2]. The mechanisms underlying EBV-mediated B cell lymphomagenesis have been studied extensively using in vitro EBV-mediated B cell immortalization as an experimental system [3]. By contrast, T/NK lymphomagenesis and epithelial carcinogenesis following EBV infection have never been reproduced in vitro. Thus, the molecular mechanisms underlying these processes remain largely unknown. 

EBV-encoded latent membrane protein 1 (LMP1) is essential for EBV-mediated B cell transformation [4]. LMP1, which belongs to the tumor necrosis factor receptor family, is a constitutively active mimic of cellular CD40 [5]. LMP1 activates downstream signaling pathways, such as NF-κB, c-jun N-terminal kinase, and p38/MAPK, through C-terminal activation regions 1, 2, 3 (CTAR1, CTAR2, CTAR3) [6,7,8], thereby triggering cell proliferation. LMP1 fulfills the criteria for a classical viral oncoprotein because its expression transforms rodent fibroblasts [9]. Numerous studies suggest that LMP1 plays a role in the pathogenesis of EBV-associated epithelial cancers [2,10]; however, due to a lack of experimental systems, the contribution of LMP1 to epithelial carcinogenesis is unclear.

EBV-encoded latent gene products, such as EBV nuclear antigens (EBNAs) and LMPs, are heterogeneous among EBV strains [11,12], and LMP1 is the most heterogeneous. LMP1 is an integral membrane protein comprising a short N-terminal cytoplasmic domain, six hydrophobic transmembrane domains, and a C-terminal cytoplasmic tail [3]. Heterogeneities reside within the six transmembrane domains as well as within the N- and C-terminal cytoplasmic domains. Heterogeneity within the C-terminal cytoplasmic domain includes variations in the copy number of repetitive 11 amino acid (a.a.) repeat motifs and a 10 a.a. deletion commonly found in EBV in Asian countries [13].

**Figure 1:**
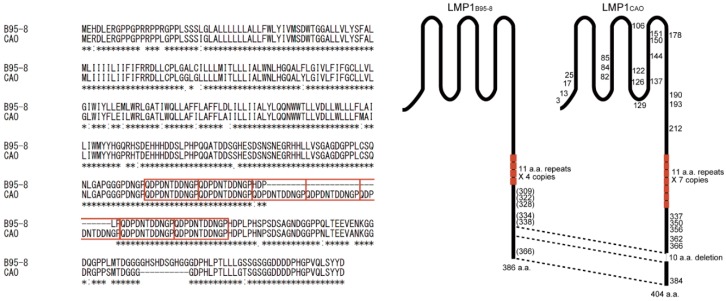
Comparison of the amino acid sequences of B95-8 LMP1 (LMP1_B95-8_) and CAO-LMP1 (LMP1_CAO_). Sequence alignment was performed by CLUSTALW (**left**); schematic illustrations of the LMP1 domains (**right**). The amino acids in LMP1_CAO_ that differ from those in LMP1_B95-8_ are shown. Note that LMP1_CAO_ contains three additional copies of 11 amino acid repeats (indicated by brown squares) and a 10 amino acid deletion in the C-terminal cytoplasmic domain.

A Chinese NPC cell CAO, propagated in nude mice, encodes LMP1 [14,15,16], designated hereafter as LMP1_CAO_, which is distinct from LMP1 expressed by prototype B95-8 strain EBV (LMP1_B95-8_) [17]. LMP1_CAO_ contains multiple a.a. substitutions within its transmembrane and N- and C-terminal cytoplasmic domains (Figure 1). It also harbors a 10 a.a. deletion in the C-terminal domain, which is common to a specific subtype of LMP1 known as China 1 type [13]. LMP1_CAO_ is functionally different from LMP1_B95-8_ in that the former is less cytostatic than the latter. This functional difference is due to the a.a. heterogeneity of the transmembrane domains rather than to the 10 a.a. deletion at the C terminus [15,16,18,19]. In addition to LMP1_CAO_, numerous NPC-associated LMP1 variants have been identified using recently developed deep sequencing technologies, and an elucidation of their roles in NPC pathogenesis is underway [20].

A previous study used a recombinant EBV (so-called ‘Maxi-EBV’ [21]) to investigate functional differences between NPC-derived LMP1 and LMP1_B95-8_ [22]. The results were somewhat unexpected: Replacing LMP1_B95-8_ with NPC-derived LMP1 decreased rather than increased the EBV copy number in latently infected cells [22]. Maxi-EBV, an EBV-BAC clone of B95-8 strain EBV, harbors a 12 kb defect in the BamHI rightward transcripts (BARTs) locus [23], which encodes a number of BART microRNAs (miRNAs). Since BART miRNAs likely contribute to EBV-mediated epithelial carcinogenesis [24], we postulated that a lack of BART miRNA genes in B95-8 EBV might hinder study of the LMP1 function.

Previously, we reported construction of BART(+) EBV-BAC, in which the BART miRNA locus of the B95-8 EBV genome fully restored [25] to allow the LMP1 function to be examined in the presence of BART miRNA. Here, we report that LMP1_CAO_ and BART miRNAs together are necessary for increased production of progeny virus from stably transfected HEK293 cells. The results suggest that LMP1 heterogeneity has a marked effect on the viral life cycle, raising the possibility that LMP1 heterogeneity contributes to EBV-mediated epithelial carcinogenesis.

## 2. Materials and Methods 

### 2.1. Cell Culture

HEK293 cells were cultured as described [25] and used for stable transfection of EBV-BAC clone DNAs and subsequent production of recombinant virus.

### 2.2. Plasmids

A nasopharyngeal carcinoma-derived *LMP1_CAO_* gene was obtained as a BamHI fragment from J124-A8-Cao5 [14] and subcloned into the pSG5 vector. The *LMP1_CAO_* gene was sequenced to verify the previously reported sequence (GenBank accession No. AF304432).

### 2.3. EBV-BAC Clones

Cloning of the B95-8 strain EBV genome and subsequent construction of BART(+) EBV-BAC were described previously [25]. The BAC clones contain a hygromycin resistance gene and a green fluorescent protein (GFP) gene as markers.

### 2.4. Engineering of the Viral Genome in E. coli

An *E. coli*-mediated genome engineering technique was used to replace the LMP1_B95-8_ gene of BART(+) EBV-BAC with LMP1_CAO_ using a positive and negative selection marker (rpsLneo), as previously described [25]. Briefly, the rpsLneo gene was PCR-amplified using primers ‘SmaI up rps’ (5-TAGGAGGGGTGGGTTCAACGCAGGGGCGTTGGTGGC

GGAGTCTGGCAACG-GGCCTGGTGATGATGGCGGGATCG-3; the sequence homologous to the rpsLneo gene is underlined) and ‘SmaI down neo’ (5-CTTTCGGGAAATCTGTACCCGTACTGCCTC

GGCAGACCCCGCAAATCCC-*GAAT-TC*AGAAGAACTCGTCAAGAAGG-3; EcoRI site in italics). The PCR product was then used to insert the rpsLneo gene into the LMP1 locus of BART(+)LMP1_B95-8_. The LMP1_CAO_ gene was PCR-amplified using primers ‘LMP1 gene up’ (5-CTCCCCTACGGTTACCCCA-3) and ‘LMP1 gene down’ (5-AGTTGTGTTGTGCAGAGGTC-3). Next, the obtained PCR product was used to replace the inserted rpsLneo gene with LMP1_CAO_. BART(−)LMP1_CAO_ was generated by deleting the 12 kb region corresponding to the B95-8-deleted region, as previously described [25].

### 2.5. Deep Sequencing

EBV-BAC clone DNAs were prepared using a NucleoBond BAC100 kit (Macherey-Nagel, Duren, Germany). Deep sequencing, which was outsourced to a service provider (Hokkaido System Science Co., Sapporo, Japan), was performed using HiSeq apparatus (Illumina, San Diego, CA, USA). The BART(+)LMP1_B95-8_ sequence [25] was used as a reference. Nucleotide mismatches were visualized using Integrative Genomics Viewer (IGV) [26]. 

### 2.6. Recombinant Virus Production and Infection

EBV-BAC clones (BART(+)LMP1_B95-8_, BART(+)LMP1_CAO_, or BART(−)LMP1_CAO_) were stably transfected into HEK293 cells, and hygromycin-resistant/GFP-positive cell clones were obtained. The cell clones were screened for their ability to produce progeny virus, and virus-producing cell clones were isolated. Progeny virus production by virus-producing cell clones was induced by transfection of BZLF1 and BALF4, as described previously [25]. Culture supernatants containing recombinant viruses were harvested at 4 days post-transfection, and the filtered supernatants were used for infection experiments. EBV-negative Akata cells [27] were used as recipient cells for infection; infection efficiency was determined by checking GFP expression by flow cytometry.

### 2.7. Expression of Viral Protein and microRNA

Expression of LMP1 was analyzed by Western blotting with anti-LMP1 monoclonal antibody S12 [28]. A TaqMan small RNA assay (Thermo Fisher Scientific, Waltham, MA, USA) was used to analyze expression of viral microRNA.

### 2.8. Reporter Assay

HEK293 cells harboring various EBV-BAC clones were transfected with pGL4.32 and pGL4.74 (Promega, Madison, WI, USA), and a Dual-Luciferase Reporter Assay was performed according to the manufacturer’s instructions.

### 2.9. LMP1 Sequences of Japanese NPC-Derived EBVs

All procedures used in this study were approved by the ethics committee of Aichi Cancer Center Research Institute (Nagoya, Japan). Biopsy specimens from three Japanese NPC patients were obtained with informed consent, and genomic DNA was isolated using a DNeasy Blood & Tissue kit (QIAGEN, Venlo, The Netherlands). The EBV LMP1 gene was PCR-amplified using ‘LMP1 gene up’ and ‘LMP1 gene down’ primers and PrimeStar MAX PCR enzyme (Takara Bio Inc., Kusatsu, Japan). The PCR products (~2150 bp in size) were subjected to DNA sequencing using ‘LMP1 gene up’, ‘LMP1 gene down’, and ‘CAOLMPseq03’ (5-CACCGTCTGTCATCGAAGGC-3) primers. Phylogenetic analyses were performed using CLUSTALW.

### 2.10. Nucleotide Sequence Accession Numbers

The patient-derived EBV LMP1 sequences can be retrieved from DDBJ/EMBL/GenBank using the accession numbers, LC467962, LC467963, and LC467964. 

## 3. Results

### 3.1. Stable Transfection of HEK293 Cells with an EBV-BAC Clone Harboring LMP1_CAO_ and BART miRNA Genes

**Figure 2:**
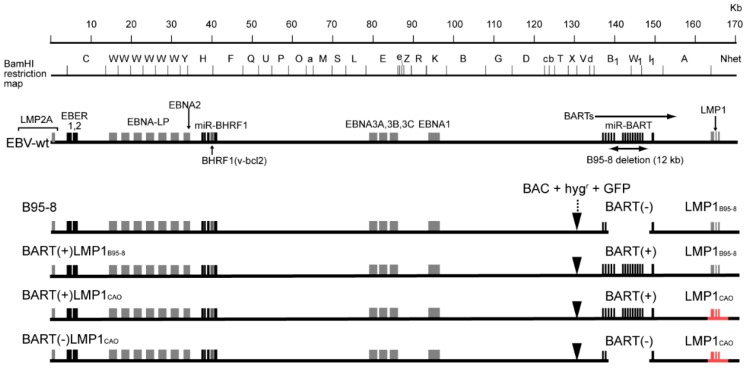
Schematic illustration of the EBV genome and four EBV-BAC clones with different BART miRNA and LMP1 statuses. The linear EBV genome, viral genes, non-coding RNAs (EBV-encoded small RNA (EBER) and BARTs), and viral microRNA genes (miR-BHRF1 and miR-BART) are illustrated. The BamHI restriction map is based on the sequence of a representative EBV strain retaining the BART miRNA locus [30]. Schematic illustration of four EBV-BAC clones (in linearized form) are shown (bottom); LMP1_CAO_ is shown in red, and the presence or absence of the BART miRNA cluster is illustrated. The insertion site for a BAC vector sequence, a hygromycin resistance gene (hyg^r^) and a GFP gene is indicated by arrowheads. DNA size is indicated at the top.

The EBV-BAC clone, ‘BART(+)LMP1_B95-8_’ [25], a derivative of the EBV-BAC clone ‘B95-8’, has a fully restored BART miRNA locus derived from Akata strain EBV [29] (Figure 2). BART(+)LMP1_B95-8_, encoding LMP1_B95-8_, was used as the starting material for this study. The LMP1 locus of BART(+)LMP1_B95-8_ EBV-BAC was replaced with a genomic DNA fragment LMP1_CAO_, which comprises a promoter region, three exons, and two introns. The resulting EBV-BAC clone, designated BART(+)LMP1_CAO_, encodes LMP1_CAO_ and has a restored BART miRNA locus (Figure 2).

**Figure 3:**
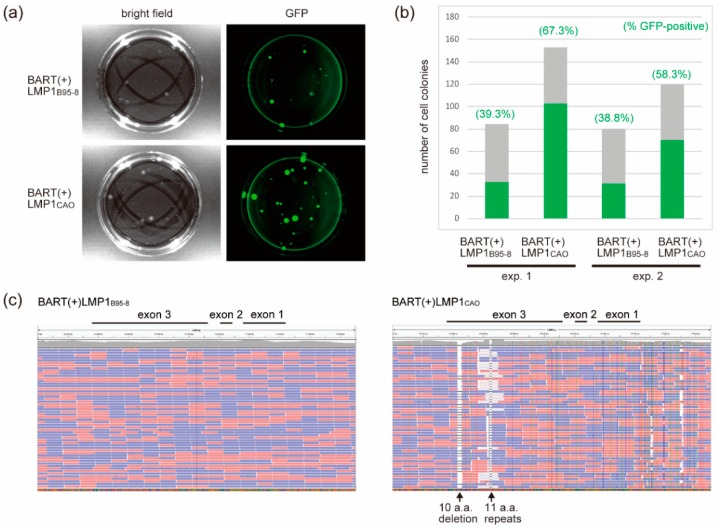
HEK293 cells stably transfected with EBV-BAC clones encoding various LMP1s. (**a**) Bright field images and GFP signals (in pseudo-color) are shown. Note that BART(+)LMP1_CAO_ generated more hygromycin-resistant/GFP-positive colonies than BART(+)LMP1_B95-8_. (**b**) The number of GFP-positive colonies was counted. The results of two independent experiments are shown. Numbers in parenthesis indicate the percentage of GFP-positive cell clones. (**c**) Nucleotide mismatches around the LMP1 locus of BART(+)LMP1_B95-8_ and BART(+)LMP1_CAO_ were visualized using Integrative Genomics Viewer (IGV). The sequence of BART(+)LMP1_B95-8_ [25] was used as a reference. Note that these BAC clones have different copy numbers of 11 a.a. repeats (Figure 1).

BART(+)LMP1_CAO_ DNA was transfected into HEK293 cells to establish virus-producing cells. BART(+)LMP1_B95-8_ DNA was used as a control. Transfected cells were re-plated into dishes at various densities and then subjected to selection with hygromycin. Hygromycin-resistant cell colonies were obtained at 3 weeks post-transfection. Representative pictures of cell colonies are shown in Figure 3a. The number of GFP-positive colonies in the dishes was counted and compiled (Figure 3b). The results revealed that BART(+)LMP1_CAO_ generated markedly more GFP-positive cell colonies than BART(+)LMP1_B95-8_ (Figure 3b).

To exclude the possibility that more efficient production of GFP-positive colonies was due to accidental mutation(s) introduced into the BAC-cloned DNA during recombinogenic engineering, we subjected DNAs from BART(+)LMP1_B95-8_ and BART(+)LMP1_CAO_ to deep sequencing. Mapping of the sequencing reads of BART(+)LMP1_CAO_ to the reference sequence (BART(+)LMP1_B95-8_) around the LMP1 locus revealed differences in the number of 11 a.a. repeats and the absence of 10 a.a. repeats (Figure 3c). However, other regions were identical in sequence (data not shown). Thus, no additional mutation was introduced into BART(+)LMP1_CAO_ during recombinogenic engineering, suggesting that LMP1_CAO_ is responsible for the increased generation of GFP-positive colonies.

### 3.2. Efficient Production of Progeny Virus by HEK293 Cells Harboring BART(+)LMP1_CAO_

**Figure 4:**
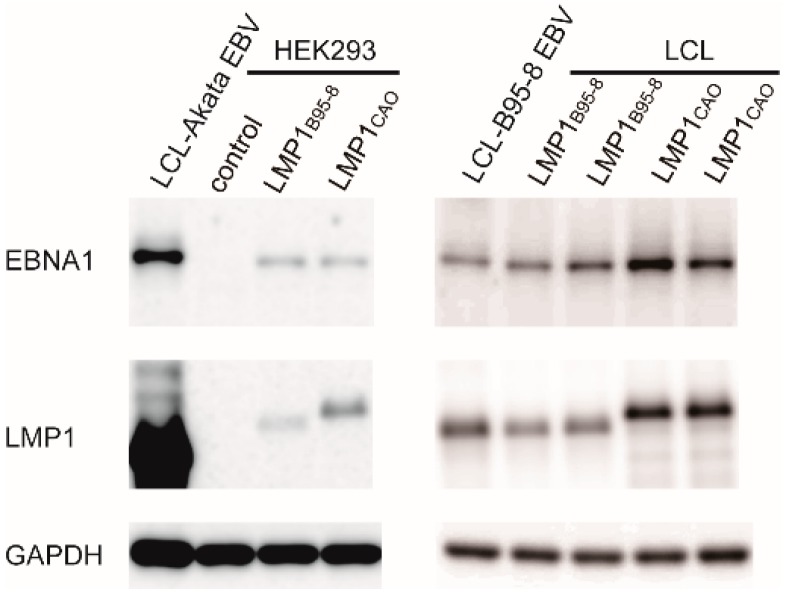
Expression of latent viral proteins by stably transfected HEK293 cells or LCLs. Expression of EBV nuclear antigen 1 (EBNA1) and LMP1 was examined by Western blot analysis. Glyceraldehyde-3-phosphate dehydrogenase (GAPDH) was used as a control.

Hygromycin-resistant, GFP-positive HEK293 cell clones stably transfected with BART(+)LMP1_CAO_ were screened for their ability to produce recombinant virus. Cell clones were transfected with a viral transactivator gene, *BZLF1* [25], and clones exhibiting high induction of viral late proteins (BALF4 and gp350/220) were chosen. Progeny viruses were obtained from selected cell clones by transfection with *BZLF1* and *BALF4* [25] and then used to infect EBV-negative Akata cells. Infection efficiency of progeny viruses was evaluated by monitoring the expression of GFP at 2 days post-infection. A typical screening result is shown in Table 1. We found that establishing virus-producing BART(+)LMP1_CAO_ cell clones was easier and more consistent than establishing BART(+)LMP1_B95-8_ cell clones. Peripheral B lymphocytes from healthy donors were infected with recombinant BART(+)LMP1_B95-8_ or BART(+)LMP1_CAO_ viruses, and lymphoblastoid cell lines (LCLs) expressing GFP were established, indicating that LMP1_CAO_ is competent for B cell transformation. Virus-producing HEK293 cells and LCLs were subjected to Western blot analyses to examine the expression of LMP1 protein. The results revealed that HEK293 cells and established LCLs expressed LMP1 proteins of the expected sizes (LMP1_B95-8_, 386 a.a.; LMP1_CAO_, 404 a.a.) (Figure 4). The expression levels of LMP1 _CAO_ were significantly higher than those of LMP1 _B95-8_ in stably transfected HEK293 cells as well as in established LCLs.

Taken together, these results indicate that the increased efficiency of BART(+)LMP1_CAO_ with respect to generating virus-producing cells is most likely due to properties intrinsic to the LMP1_CAO_ protein, which are distinct from those of LMP1_B95-8_.

### 3.3. Deletion of BART miRNA Compromises the Ability to Generate Progeny Viruses

**Figure 5:**
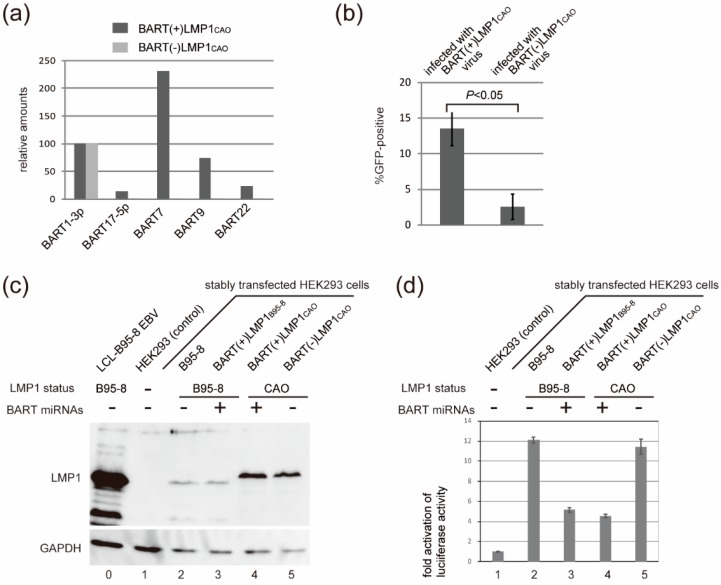
The effect of BART miRNA deletion on progeny virus production and NF-κB signaling activity. (**a**) Loss of BART miRNA expression from HEK293 cells harboring BART(−)LMP1_CAO_ was verified by a PCR-based assay. The amounts of mature BART1-3p miRNA, whose gene is located outside of the BART deletion, are presented as 100. (**b**) Progeny viruses of BART(+)LMP1_CAO_ or BART(−)LMP1_CAO_ were used to infect EBV-negative Akata cells. The percentage of GFP-positive cells was measured by fluorescence activated cell sorting analysis. (**c**) LMP1 expression levels in stably transfected HEK293 cells harboring EBV-BAC clones as indicated (lanes 2 through 5). Lane 0; LCL established by B95-8 strain EBV. Lane 1; control HEK293 cell. (**d**) The NF-κB reporter plasmid was used to examine NF-κB signaling activities in HEK293 cells harboring various EBV-BAC clones. Lane numbers correspond to those in (**c**).

Next, we examined whether BART miRNAs are necessary for efficient progeny virus production of BART(+)LMP1_CAO_. For this purpose, a BAC clone of BART(+)LMP1_CAO_ was subjected to recombinogenic engineering to obtain BART(−)LMP1_CAO_ (Figure 2). HEK293 cells were transfected with either BART(+)LMP1_CAO_ or BART(−)LMP1_CAO_, and the efficiency with which virus-producing cells were obtained was compared. Loss of BART miRNA expression in HEK293 cells harboring BART(−)LMP1_CAO_ was verified using a PCR-based assay (Figure 5a). Four independent cell clones harboring either BART(+)LMP1_CAO_ or BART(−)LMP1_CAO_ were established. The levels of viral late proteins (BALF4 and gp350/220) following BZLF1 transfection were markedly higher in BART(+)LMP1_CAO_-transfected cells than in BART(−)LMP1_CAO_-transfected cells (data not shown). Progeny viruses obtained from selected cell clones harboring either BART(+)LMP1_CAO_ or BART(−)LMP1_CAO_ were used to infect EBV-negative Akata cells. The results revealed that the infection efficiency of BART(−)LMP1_CAO_ viruses was significantly impaired compared with that of BART(+)LMP1_CAO_ viruses (Figure 5b). Thus, LMP1_CAO_ and BART miRNAs together are necessary for efficient production of progeny virus in stably transfected HEK293 cells.

We next investigated the mechanism via which LMP1_CAO_ and BART miRNA enable efficient progeny virus production. HEK293 cells stably transfected with each of four EBV-BAC clones (Figure 2) were subjected to Western blot analyses and NF-κB reporter assays. Western blot analyses revealed that, as previously noted (Figure 4), the expression levels of LMP1_CAO_ were significantly higher than those of LMP1_B95-8_, regardless of BART miRNA status (Figure 5c). On the other hand, the results of the NF-κB reporter assay clearly indicated that having the intact BART miRNA locus resulted in the attenuation of NF-κB signaling activity. Specifically, HEK293 cells harboring BART-negative EBV-BAC clones exhibited stronger NF-κB signaling activity (Figure 5d, lanes 2 and 5), while HEK293 cells harboring BART-positive EBV-BAC clones exhibited weaker activity (Figure 5d, lanes 3 and 4). Differences of the LMP1 status (either LMP1_B95-8_ or LMP1_CAO_) did not markedly affect NF-κB signaling activity. Thus, there was no correlation between the efficiency of progeny virus production and NF-κB signaling activity (compare Figure 5b,d). Similar results were obtained using another set of four HEK293 cell clones harboring each of the four EBV-BAC clones (data not shown).

Thus, we conclude that LMP1_CAO_ and BART miRNAs together are necessary for efficient progeny virus production, and that the mechanism underlying the phenomenon does not involve activation of the NF-κB signaling pathway.

### 3.4. Amino Acid Sequences of LMP1 from Japanese NPC Patients 

**Figure 6:**
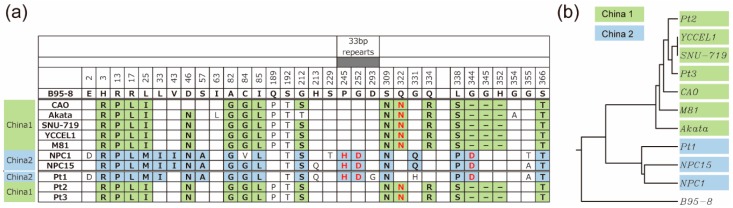
LMP1 amino acid sequences of EBV strains derived from Japanese NPC patients. (**a**) Varying amino acid sequences among LMP1 derived from Japanese EBV strains from NPC patients (Pt1, Pt2, and Pt3) and other representative EBV strains. Numbers correspond to amino acid positions within LMP1_B95-8_. Amino acid changes are denoted by letter changes; a dash represents a deletion. Strains harboring amino acid changes are shown in bold and shaded in different colors. Unique amino acids found in China 1 or China 2 are shown in red. The position of the 33 bp repeats is denoted at the top. LMP1 sequences were retrieved from GenBank. Accession numbers are as follows: B95-8, V01555; CAO, AF304432; Akata, KC207813; SNU-719, AP015015; YCCEL1, AP015016; M81, KF373730; NPC1, EF419184; NPC15, AY601321; Pt1, LC467962; Pt2, LC467963; Pt3, LC467964. (**b**) Phylogenetic tree constructed from the LMP1 amino acid sequences of Japanese NPC-derived EBV strains and other representative EBV strains.

Previous studies indicate that the LMP1 genes of various EBV strains can be grouped according to changes in signature a.a. [13]. LMP1_CAO_ is a China 1 type LMP1; this group is prevalent in Asian EBV samples [13,31] (Figure 6a). Similarly, LMP1 from Akata (Japanese Burkitt lymphoma-derived) [29], SNU-719 and YCCEL1 (both Korean gastric cancer-derived) [32,33], and M81 (Chinese NPC-derived) [34] belongs to China 1 type. Here, we sequenced EBV LMP1 derived from three Japanese NPC patients (LMP1_Pt1_, LMP1_Pt2_, and LMP1_Pt3_) to examine LMP1 heterogeneity. LMP1_Pt1_ does not harbor a 10 a.a. deletion in its C-terminal cytoplasmic domain, whereas LMP1_Pt2_ and LMP1_Pt3_ do. LMP1_Pt1_ belongs to China 2 type as it encodes a histidine residue at position 245 and aspartic acid residues at positions 252 and 344 (Figure 6a). By contrast, LMP1_Pt2_ and LMP1_Pt3_ belong to the China 1 type. Phylogenetic analyses supported the idea that LMP1_Pt2_ and LMP1_Pt3_ resemble LMP1_CAO_ and other China 1 type LMP1, whereas LMP1_Pt1_ was more closely related to China 2 type LMP1, such as NPC1 and NPC15 [35] (Figure 6b). These results are compatible with those of a previous report showing that China 1 type LMP1 is predominant among EBV strains isolated from Japanese individuals [36].

## 4. Discussion 

This study compared the behavior of NPC-derived LMP1 (LMP1_CAO_) with that of LMP1_B95-8_ in the context of the EBV genome. A previous study used a ‘Maxi-EBV’ [21], which lacked the majority of BART miRNAs, to answer the same question [22]. The results of that study showed that replacing the LMP1_B95-8_ gene with NPC-derived LMP1, which is similar, but not identical to LMP1_CAO_, led to a marked reduction in the EBV copy number in stably transfected HEK293 cells. By contrast, the present study used a recombinant EBV in which the BART miRNA locus was fully restored [25]. We found that replacing the LMP1_B95-8_ gene with the LMP1_CAO_ transgene led to a marked increase in the number of virus-producing cells generated. However, the effect was observed only in the presence of the BART miRNA locus. Thus, the BART miRNA locus is a prerequisite for efficient establishment of virus-producing cells. Enhanced progeny virus production increases the probability of epithelial cell infection and tumorigenesis. We observed that LMP1_CAO_ expression was higher than that of LMP1_B95-8_ in stably transfected HEK293 cells and in LCLs. Since the cytostatic activity of LMP1_CAO_ is lower than that of LMP1_B95-8_ [15,16,18,19], cells with increased LMP1_CAO_ expression are likely to be selected. We speculate that increased LMP1 expression, together with BART miRNA–mediated attenuation of NF-κB signaling activity, enables efficient progeny virus production. Increased LMP1 expression could also contribute to epithelial tumorigenesis, as LMP1 plays pivotal roles in tumorigenesis [2,10].

A few studies have focused on the functional interaction between LMP1 and BART miRNAs. For example, a previous study demonstrated that BART miRNAs modulate expression of LMP1 protein [37]. Another study suggested the presence of a negative feedback loop, in which upregulation of the BART promoter by LMP1 leads to an increase in BART miRNA expression that then downregulates the LMP1 protein level [38]. A study utilizing EBV reverse genetics demonstrated that a BART miRNA knockout virus induces more rapid cell growth in humanized mice, probably due to increased expression of LMP1 [39]. In our in vitro cell culture system, LMP1 expression levels were not affected by deletion of BART miRNA genes. Whether or not LMP1 and BART miRNAs functionally interact with each other remains to be investigated.

The mechanism via which LMP1_CAO_ and BART miRNAs enable increased progeny virus production remains to be clarified. We found that the mechanism does not involve the activation of the NF-κB signaling pathway. Our data indicate that BART miRNAs downregulate the NF-κB signaling pathway, but not through the downregulation of LMP1. This clearly contradicts the results of a recent report showing that a BART miRNA (miR-BART13) upregulates NF-κB signaling activity by targeting an inhibitor of the NF-κB signaling pathway [40]. This inconsistency should be clarified in future studies. Since BART miRNAs target a variety of cellular and viral genes, we suggest that BART miRNA-mediated regulation of global gene expression networks likely underlies its positive effect on progeny virus production in EBV-infected cells.

We also constructed another EBV-BAC clone harboring LMP1 from Akata strain EBV. This BAC clone was subjected to the same experiments described above. The results indicated that the colony forming efficiency of BART(+)LMP1_Akata_ was, like that of BART(+)LMP1_CAO_, superior to that of BART(+)LMP1_B95-8_ (data not shown). Thus, LMP1_Akata_ and LMP1_CAO_, both of which belong to the China 1 group [13], share similar biological activities. It is unclear whether China 2 group LMP1 is functionally distinct from China 1 group LMP1.

The EBV-BAC clone ‘BART(+)LMP1_CAO_’ appears to be an ideal starting material for EBV genetics. A major technical difficulty with the EBV-BAC system is the poor efficiency in obtaining cells that produce high-titer virus [41]. Here, we obtained clear evidence that it is much easier to obtain virus-producing cells using BART(+)LMP1_CAO_ than BART(+)LMP1_B95-8_. From a practical point of view, it is very important to use BART(+)LMP1_CAO_ because it accelerates production of various recombinant viruses.

Future experiments should focus on clarifying the mechanism(s) of EBV-mediated epithelial carcinogenesis, to which NPC-derived LMP1 likely contributes. Our data show that replacing a single critical viral gene has a significant impact on the viral biological function. This highlights the importance of using appropriate viral strains when studying epithelial carcinogenesis. For example, to study carcinogenesis of the gastric epithelium, it is most appropriate to use a combination of gastric cancer-derived EBVs and gastric epithelial cells. A recent study also highlights the importance of using appropriate EBV strains for studying specific types of EBV-mediated tumorigenesis [42]. Recently, we developed a method based on the CRISPR/Cas9-mediated genome editing technology and used it to rapidly clone EBV genomes from EBV-positive gastric cancer cell lines [30]. The same experimental strategy can be applied to various EBV-associated malignancies. Examining the biological properties of various cancer-derived EBVs is important if we are to clarify how different EBV strains influence EBV-mediated tumorigenesis.

## Figures and Tables

**Table 1:** Virus-producing cell clones obtained by transfection of EBV-BAC clones into HEK293 cells.

HEK293 Cells Transfected with	BART(+)LMP1_B95-8_	BART(+)LMP1_CAO_
No. of cell clones screened	16	28
No. of virus-producing cell clones obtained	2	5
infection efficiencies of progeny viruses to B cell lines	10.0% 15.6%	10.5% 11.5% 15.3% 16.5% 29.2%
